# Current status of ctDNA in precision oncology for hepatocellular carcinoma

**DOI:** 10.1186/s13046-021-01940-8

**Published:** 2021-04-26

**Authors:** Yan Li, Yuanyuan Zheng, Liwei Wu, Jingjing Li, Jie Ji, Qiang Yu, Weiqi Dai, Jiao Feng, Jianye Wu, Chuanyong Guo

**Affiliations:** 1 Department of Gastroenterology, Putuo People’s Hospital, Tongji University School of Medicine, number 1291, Jiangning road, Putuo, Shanghai, 200060 China; 2 Department of Gastroenterology, Shanghai Tenth People’s Hospital, Tongji University School of Medicine, Number 301, Middle Yanchang road, Jing’an, Shanghai, 200072 China

**Keywords:** ctDNA, Hepatocellular carcinoma, Mutation, Methylation

## Abstract

The conventional method used to obtain a tumor biopsy for hepatocellular carcinoma (HCC) is invasive and does not evaluate dynamic cancer progression or assess tumor heterogeneity. It is thus imperative to create a novel non-invasive diagnostic technique for improvement in cancer screening, diagnosis, treatment selection, response assessment, and predicting prognosis for HCC. Circulating tumor DNA (ctDNA) is a non-invasive liquid biopsy method that reveals cancer-specific genetic and epigenetic aberrations. Owing to the development of technology in next-generation sequencing and PCR-based assays, the detection and quantification of ctDNA have greatly improved. In this publication, we provide an overview of current technologies used to detect ctDNA, the ctDNA markers utilized, and recent advances regarding the multiple clinical applications in the field of precision medicine for HCC.

**Supplementary Information:**

The online version contains supplementary material available at 10.1186/s13046-021-01940-8.

## Background

Hepatocellular carcinoma (HCC) is a malignant tumor with high morbidity and mortality worldwide, ranking sixth in incidence and fourth in cancer-related mortality [1, 2]. Many patients are at an advanced stage at the time of diagnosis, thus losing the possibility for curative surgery. Patients with early-stage HCC (BCLC stage A) who undergo radical surgical resection or ablation still have a 50–70% recurrence rate [3]. Thus, early diagnosis and precise and timely implementation of therapeutic agents are key steps by which to improve prognosis.

The early disseminated recurrence of HCC is mainly evaluated by serum alpha-fetoprotein (AFP) levels, imaging studies, and tissue biopsies. Currently, screening of patients with HCC mainly relies on the serum AFP and ultrasound of the liver, the combination of which has a sensitivity of only 63% [4]. In addition, imaging and tumor biopsy have limited diagnostic potential and sensitivity. For example, ultrasound is an operator-dependent modality [5] and some lesions are difficult to access. Moreover, the molecular pathogenesis is highly heterogeneous and complex in HCC, and the information provided from a single biopsy always fails to reflect the heterogeneity. Therefore, it is imperative to create an efficient method to detect early HCC and to guide precise management of the cancer.

**Fig. 1:**
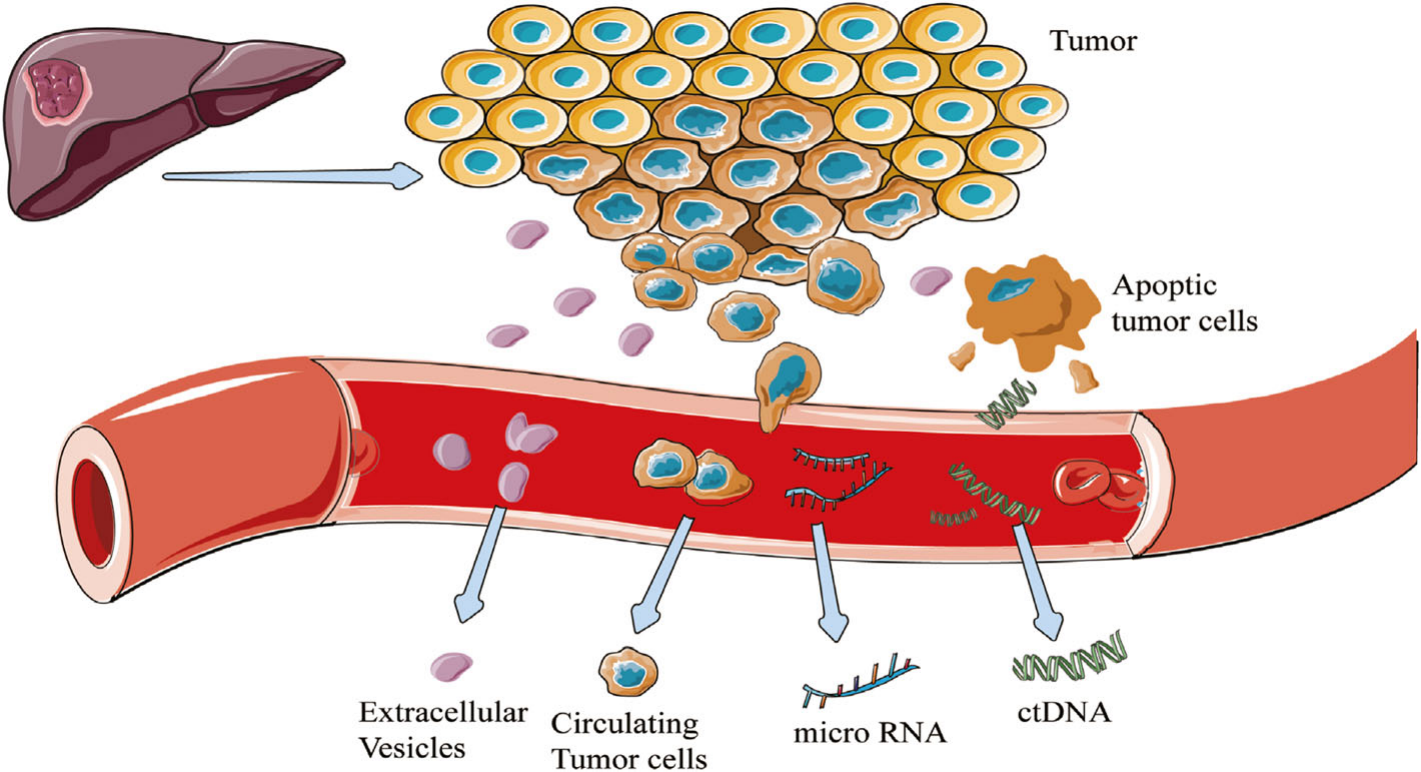
Illustration of common liquid biopsy markers:circulating tumor DNA (ctDNA), microRNA (miRNA), and extracellular vesicles (EVs)

The analytes of liquid biopsy, such as circulating tumor cells (CTCs), circulating tumor DNA (ctDNA), microRNA (miRNA), extracellular vesicles (EVs), proteomics, metabolomics, and transcriptomics have shown the potential to overcome these limitations. Each of those analytes targeted by the liquid biopsy has advantages and limitations in addressing clinical needs (Table 1). Specifically, ctDNA is a cornerstone of a liquid biopsy. The term ctDNA refers to the approximately 150–200 base fractions of total circulating-free DNA (cfDNA), which originates from tumor cells (Fig. 1) [6]. There are multiple mechanisms that have been postulated for the release of ctDNA by apoptosis or necrosis of tumor cells or products from macrophages that have phagocytized necrotic tumor cells [7].

**Table 1:** Comparison of advantages and limitations of analytes found in liquid biopsy samples

	Advantages	Limitations
CTCs	Available for analysis of splice variants, information at single-cell level and functional assays regard to genomics, transcriptomics, metabolomics and proteomics Capable of subsequent culture and further biological analyses Useful for screening new drug, drug resistance and treatment test Identifying tumor patients with minimal residual disease who are at risk of recurrence	Low abundance in biofluids and difficult in capture and isolation Lack of consensus on isolation and detection methods makes comparison of data from different platforms challenging Expression loss of epithelial cell surface markers during the epithelial-to-mesenchymal transition process High degree of heterogeneity
ctDNA	Providing a comprehensive overview of genomic spectrum respond to different regions of the tumor Improvements in technology enabled greater sensitivity of analytical assay Short half-life of ctDNA allowing for real-time monitoring of cancer More precise with respect to clinical correlations	Time-consuming and highly cost Most of the emerging assays have not yet been clinically validated Genetic information only, not information on the body site of the cancer concerned
miRNA	With broad application prospects because of miRNAs are involved in many pathogenic processes High specificity and reproducibility A good candidate for cancer prevention because of patients with precancerous lesions also showing an altered pattern of circulating miRNAs	The rupture of erythrocytes and platelet containing miRNAs may influence detection levels during sample extraction and preparation Co-morbidities can lead to increased miRNAs and interfere with the detection of cancer-specific miRNA levels Technical limitation
cfRNA	Capable to present the up-to-date snapshot of the transcriptome Can be used to differentiate cancer subtypes Be able to detect cancer and trace it back to its origin site	Lack of robustly designed and independently validated biomarker studies. Low quantity and low quality in biofluids High variability of cfRNA expression between individuals
EVs	Carrying multiple biological information released from parent cells, including proteins, nuclear acids, lipids and metabolites and capable to provide information exchange EVs are more abundant in plasma/serum compared to CTCs and much more stable in circulation by protection of a lipid membrane compared with cfDNA	Small size and low density make isolation and analysis difficult High transport and collection requirements Being interfered by co-morbidities or medical therapy background
Circulating proteins	Initial attempts to combine circulating proteins with other analytes was suggested to improve early detection of cancer	Only a small number of established protein markers have been applied in clinics Information about tissue specificity or cancer specificity is largely missing Very low abundance, high complexity and dynamic nature involved
Metabolites	Providing an overview of the physiological state connected with the phenotype Potentially for differentiating between benign and malignant lesions	Technical limitations Few relevant studies

*Abbreviation*: *CTCs* circulating tumor cells, *ctDNA* circulating tumor DNA, *cfDNA* cell free DNA, *EVs* extracellular vesicles, *cfRNA* cell free RNA

A number of studies have focused on ctDNA as a novel biomarker for early diagnosis, surveillance for recurrence or progression, and prognostication in several common malignancies [8–11]. In our review, we have outlined the current status of ctDNA detection, marker selection, and emphasize the strong role of ctDNA in precision medicine for HCC.

## Current methodologies for detection of ctDNA

Ultrasensitive technology for detection of ctDNA is needed considering that ctDNA is highly fragmented by nature and diluted among overall cfDNA in patients, especially for patients with early-stage cancer who have a light tumor burden, presenting < 0.1% according to previous studies [12–14].

### Pre-analytical analysis

Appropriate sample preparation can significantly lower the false-negative rate related to a low amount of ctDNA input for numerous assays. Conventional approaches involve complicated sample preparation steps, including sample collection, matrix selection, conservation, thawing, sample processing, and ctDNA extraction. The interval between venipuncture and sample processing should be short, while a specialized blood collection tube containing a preservative, such as Streck (Streck, Omaha, NE) or PAXgene (PreAnalytiX GmbH, Hombrechtikon Switzerland) blood collection tubes, make samples stable [15–17]. Furthermore, a storage temperature up to − 80 °C is required and < 3 freeze and thaw cycles are recommended [18]. During sample processing, a two-step high-speed centrifugation is always performed [16]. Protocols that are created for ctDNA extraction based on spin column, magnetic bead, and phase isolation, vary with plasma volumes and extraction methods. There are commercial kits, such as the QIAamp Circulating Nucleic Acid™ kit (Qiagen, Germany) have been applied [19].

New technologies for the isolation of ctDNA from background contaminates have improved the potency of pre-analytic procedures, especially microfluidics and nanotechnology. For example, Sonnenberg et al. [20] proposed a dielectrophoresis-based microarray device that separates cfDNA into the microelectrodes embedded in high-field regions and performs detection of the concentrated cfDNA on-chip by fluorescence. Lee et al. [21] also reported nanochip- and nanowire-based assays, capturing the ctDNA by switching the oxidation state of the conducting polymer followed by release. Therefore, the research approaches allow for better cfDNA yields, efficient processing, and less loss and damage during extraction.

### Analytic approaches

Currently, ctDNA analysis is generally performed using PCR-based methods, including real-time PCR, droplet digital PCR (ddPCR), beads, emulsion, amplification, and magnetics (BEAMing), as well as sequencing-based technologies.

A range of real-time PCR-based assays have been designed for the detection of targeted SNV in ctDNA [22–24]. Although the traditional assays are easy to operate and cost-effective, most of the traditional assays have low sensitivity and a limited number of targeted loci can be analyzed. Advances in co-amplification at lower denaturation temperature (COLD)-PCR [25], bidirectional pyrophosphorolysis-activated polymerization (bi-PAP) [26], and Intplex [27–29] were developed for analysis of low-abundance mutations.

**Fig. 2:**
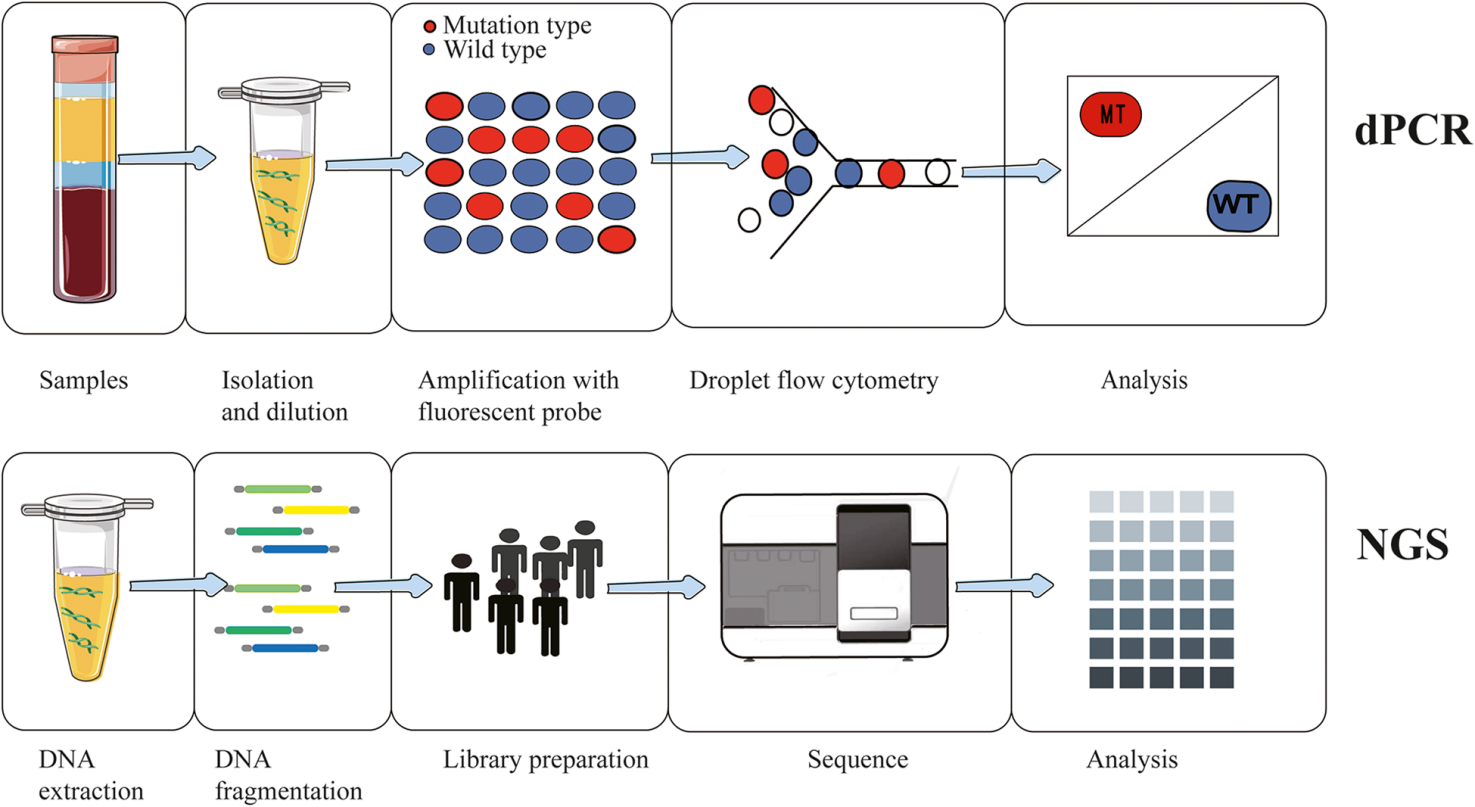
Schematic workflow of droplet-based digital PCR (dPCR) and next-generation sequencing (NGS)**.** dPCR consists of sample isolation, limited dilution, separations of a single sequence into each microcompartment, individual amplification, and immunofluorescence staining for specific sequence. NGS method profiles target genes by fracturing the DNA molecules into small sequences and proceeds with massive parallel sequencing

Digital PCR was defined as one of the standard reference methodologies for the analysis of ctDNA with high sensitivity and the ability to quantify results. The basic workflow schematic is depicted in Fig. 2. The dPCR assay allows the identification of rare target-mutated genes by compartmentalization and amplification. The method consists of limited dilution, separations of a single sequence into each microcompartment, individual amplification, and immunofluorescence staining for specific sequences. Droplet digital PCR (ddPCR) involves millions of monodisperse droplets generated by microfluidic emulsification to create PCR microreactors that can perform millions of reactions in parallel [30, 31]. Another method with high-resolution detection (beads, emulsion, amplification, and magnetics [BEAMing]) can detect mutations as rare as 0.01% [32]. In the process, the single magnetic bead tethered with a starting DNA template is partitioned into each water-in-oil microemulsion, where thousands of amplification reactions are subsequently performed. Then, the beads are purified and hybridized with allele-specific fluorophore probes to discriminate mutant genes from wild types [33–35].

In contrast to dPCR methodology screening for pre-defined variants, NGS-based technologies make entire genome sequencing feasible and allow for detection of non-hotspot relevant mutation sequences. With the advent of NGS, every unknown mutation and emergent cancer-related genetic alterations during the tumor evolution period can be profiled by fracturing the DNA molecules into small sequences and massive parallel sequencing of multigenes [36, 37]. Frequently-applied assays, such as tagged-amplicon deep sequencing (Tam-Seq) [37], a safe-sequencing system (Safe-SeqS) [38], cancer personalized profiling by deep sequencing (CAPP-Seq) [39], and ion torrent [40], permitted sequencing of multiple targets. As for non-target variants, NGS technology, such as whole-exome sequencing (WES) [41], whole-genome sequencing (WGS) [42], and Methyl-Seq [43, 44], are also available for genome-wide sequencing. Nevertheless, NGS-based genome-wide ctDNA analyses have a higher requirement for ctDNA concentration and a sensitivity of 1–5%, making NGS-based genome-wide ctDNA analyses unsuitable for monitoring residual disease before disease relapse [45].

In addition to NGS-based untargeted sequencing, personalized analysis of rearranged ends (PARE) is a PCR-based approach allowing for untargeted identification of cancer-specific rearrangements in ctDNA, while digital karyotyping provides untargeted information of chromosomally-changed genomes or new genomic regions [16, 46].

The aforementioned technologies are limited by complicated sample preparation and interference from bioenvironmental components. Thus, many advanced technologies have been developed for ultrasensitive detection of multiplex minor variants without those limitations. Plasmonic nanoparticles are used in surface-enhanced Raman scattering (SERS) nanosensors for signal-amplification and mutations are identified based on specific Raman spectroscopy [47, 48]. For matrix-assisted laser desorption/ ionization time of flight mass spectrometry (MALDI-TOF-MS), biotin-labeled extended products are captured and eluted, then dispensed onto bioarrays for spectrum profiles [49]. Relatively, the electrochemical biosensors are more widespread and easier to fabricate, time- and cost-effective, rapidly responsive, and portable. The device incorporates immobilized DNA as a molecular recognition element on the electrode surface. The introduction of nanostructured materials as an interfacial film enables improved recognition capability and increased signal output intensity [50, 51]. A summary and comparison of all these technologies are shown in Table 2. Despite all advantages, these devices are not widely used in clinics. Perhaps there is still a gap in the translation from laboratory prototypes to clinical devices or there is a reluctance from users to this new and unfamiliar technology.

**Table 2:** Summary of analytical approaches for ctDNA detection

Assay		Principle	Type of alteration	Limit of detection (mutant allele frequency)	DNA input	Evaluation	Reference
Real-time PCR		PCR primers with 3′ nucleotide extension utilizing mutated target genes	Known mutations	10–20%	2 ml of plasma	Easy to perform Qualitative analysis Unable to dynamic monitoring of cancer	[23, 24]
COLD-PCR		Utilizing the threshold temperature in the PCR, wild-type mutant heteroduplexes are selectively denatured to enrich for rare mutations	Known mutations	0.01%	25 pg-25 ng	Short time to output Enrich rare mutations Semi-quantitative	[25]
Bi-PAP		Primers with an overlapping nucleotide at the 3′ end activate the pyrophosphorolysis upon binding to the cognate template, thus allowing strand extension	Known mutations	0.01%	2 ml of plasma	Cost-effective Time-waste High error rate	[26]
Intplex		Mutant-specific primers are hybridized with a blocking oligonucleotide containing a phosphate group at the 3′ end to block the extension of the wild-type sequence	Known mutations	0.004%	2.25 pg/ml	Cost-effective Rapid data turnaround Pre-knowledge of genetic variants	[28, 29]
dPCR based	ddPCR	Involves millions of monodisperse droplets generated by microfluidic emulsification to create PCR microreactors that can perform millions of reactions in parallel	Known mutation	0.001%	5 ng/per reaction	Input amount depended sensitivity Easy to perform Pre-knowledge of genetic and epigenetic variants	[30, 31]
	BEAMing	Involves inputting pre-amplified products with primer-coated beads into limiting dilutions and performing further PCR reactions before the beads are purified and ligated to allele-specific fluorophore probes to distinguish between mutant and wild-type DNA	Known mutation	Less than 0.01%	2 ml of plasma	High sensitivity Low sequencing cost Rapid when compared to NGS Pre-knowledge of genetic and epigenetic variants	[32–35]
NGS based	TAm-Seq	Flexibly adapted to sequence multiple interested genomic regions in parallel by designing primers to amplify short amplicons	SNVs/indels/CNVs	0.02%	1 ml	Cost- and time effective High throughput Higher error rate	[37]
	Safe-SeqS	Tags each template DNA with unique molecular identifiers prior to amplification to create a unique family of sister molecules descended from the same original molecule	SNVs/indels	0.1%	3 ng	Improve the accuracy of massively parallel sequencing limited by the fidelity of the polymerase used in the PCR step	[38]
	CAPP-Seq	Relied on a multiphase bioinformatics workflow to devise a “selector” for subsequent capture and sequence of mutated regions of interest	SNVs/indels/CNVs /Rearrangements	0.02%	32 ng	Low sequencing cost High coverage Improved Sensitivity Sequencing artifacts	[39]
	Ion Torrent	Relies on standard DNA polymerase sequencing with unmodified dNTPs but uses semiconductor-based detection of hydrogen ions released during every cycle of DNA polymerization	SNVs/indels /CNVs/ fusions	0.1%	20 ng	Low sequencing cost High error rate	[40]
	Methyl-Seq	Based on affinity, restriction enzyme or bisulfite conversion and utilize microarray or sequencing platforms downstream	Methylated regions	–	~ 50 ng	Genome-wide coverage Bisulfite treatment damages the DNA	[43, 44]
	WES	Amplification and sequence of the whole exome regions	SNVs/indels	More than 5–10%	25 ng	Huge amounts of data per sample Low depth of coverage	[41]
	WGS	Amplification and sequence of the whole genome regions	CNVs/SVs	–	5-10 ng	High depth of coverage Costly	[42]
SERS		Multiplex mutation-specific primers amplify tumor DNA, followed by labeling of amplicons with specific SERS nanotags and enrichment with magnetic beads. Afterwards, Raman detection was performed to identify the mutations	SNVs	0.1%	2 ng/ul	Ultrasensitive Portable Bias in signal detection process Not yet applied in clinics	[47, 48]
MALDI-TOF-MS		Composed of multiplex PCR and mutation-specific single-base extension reactions while mutational genotypes are identified and characterized using matrix-assisted laser desorption/ionization time- of-flight mass spectrometry	SNVs	Less than 0.1%	~ 10 ng	Multiple targets Ultrasensitive Unlimited sample throughput Few relevant studies on ctDNA	[49]
Electrochemical biosensor		The device incorporates immobilized DNA as a molecular recognition element on the electrode surface and with the introduction of nanostructured materials as interfacial film	SNVs	0.01%	12.5 k copies/μl or 20 ng in 10 μl	Time and cost-effective Rapid response Portability Not yet applied in clinics	[50, 51]
PARE		Biotin labels tag the ends of template sequences and then mate pairs are analyzed to identify intra-and inter-chromosomal rearrangements.	Genome-wide rearrangements	0.001%	–	Whole genome coverage False-negative results	[46]
Digital karyotyping		Short genomic DNA tags were concatenated, cloned, and sequenced	chromosomally changed genomes/ new genomic regions	–	–	Rare clinical trials	[16, 46]

*Abbreviations*: *ctDNA* circulating tumor DNA, *PCR* polymerase chain reaction, *SNV* single nucleotide variation, *CNV* copy number variation, *SV* structural variation, *Bi-PAP* bidirectional pyrophosphorolysis-activated polymerization, *COLD* Co-amplification at lower denaturation temperature, *Tam-Seq* Tagged-amplicon deep sequencing, *Safe-SeqS* Safe-Sequencing System, *CAPP-Seq* Cancer Personalized Profiling by deep sequencing, *WES* whole-exome sequencing, *WGS* whole- genome sequencing, *SERS* surface-enhanced Raman scattering, *MALDI-TOF-MS* matrix-assisted laser desorption/ ionization time of flight mass spectrometry, *PARE* personalized analysis of rearranged ends

### Available guidelines

Various pre-analytic factors, such as time interval and temperature of biofluids before purification, storage temperature, collection tubes, relevant stabilization reagents, and extraction protocols, can result in variable DNA yields, sequence bias, sample contaminations, and DNA degradation [52]. During the subsequent analysis, amplification bias, sequencing artefacts, and adoption of different laboratory techniques can all influence the final results. Thus, establishing a standard operating procedure (SOP) and strict quality control are of great significance in increasing the validity and comparability of ctDNA analysis results.

Many efforts were paid to construct a unified SOP. For ctDNA isolation, the European Committee for Standardization (CEN), as part of the Standardization of generic Pre-analytical procedures for In-vitro DIAgnostics for Personalized Medicine (SPIDIA) program, have proposed recommended guidelines for sample preparation (ISO 20186-3:2019 document [https://standards.cen.eu/]). The European Consortium Cancer ID (https://www.cancer-id.eu) and the United States Working Groups Blood Profiling Atlas in Cancer (BloodPAC [https://www.bloodpac.org/]) published best-practice protocols when implementing liquid biopsies [53]. Additionally, before NGS-based technologies are applied, full validation, cyclic testing, and external quality assessment should be done according to ISO 15189 [54].

## Molecular profiling of ctDNA for HCC

### Hot-spot mutated genes in ctDNA

Understanding the molecular features of the pathogenesis underlying HCC can shed light on the development of targeted agents in HCC. The majority of approved agents are angiogenesis inhibitors and targeting multiple tyrosine kinase receptors, such as VEGFR and PDGFR [55]. Sorafenib was the first molecular medicine approved, while lenvatinib, regorafenib, and cabozantinib have also received approval recently. Active agents blocking immune checkpoint programmed cell death protein 1 (PD1) or its ligands (PDL1) have also been approved as anchor drugs, including ramucirumab, nivolumab, and pembrolizumab [56]. Despite this, therapeutic applications of target drugs derived from genomic alterations are still slow to be adopted and worthy of further investigation at the genomic level.

**Fig. 3:**
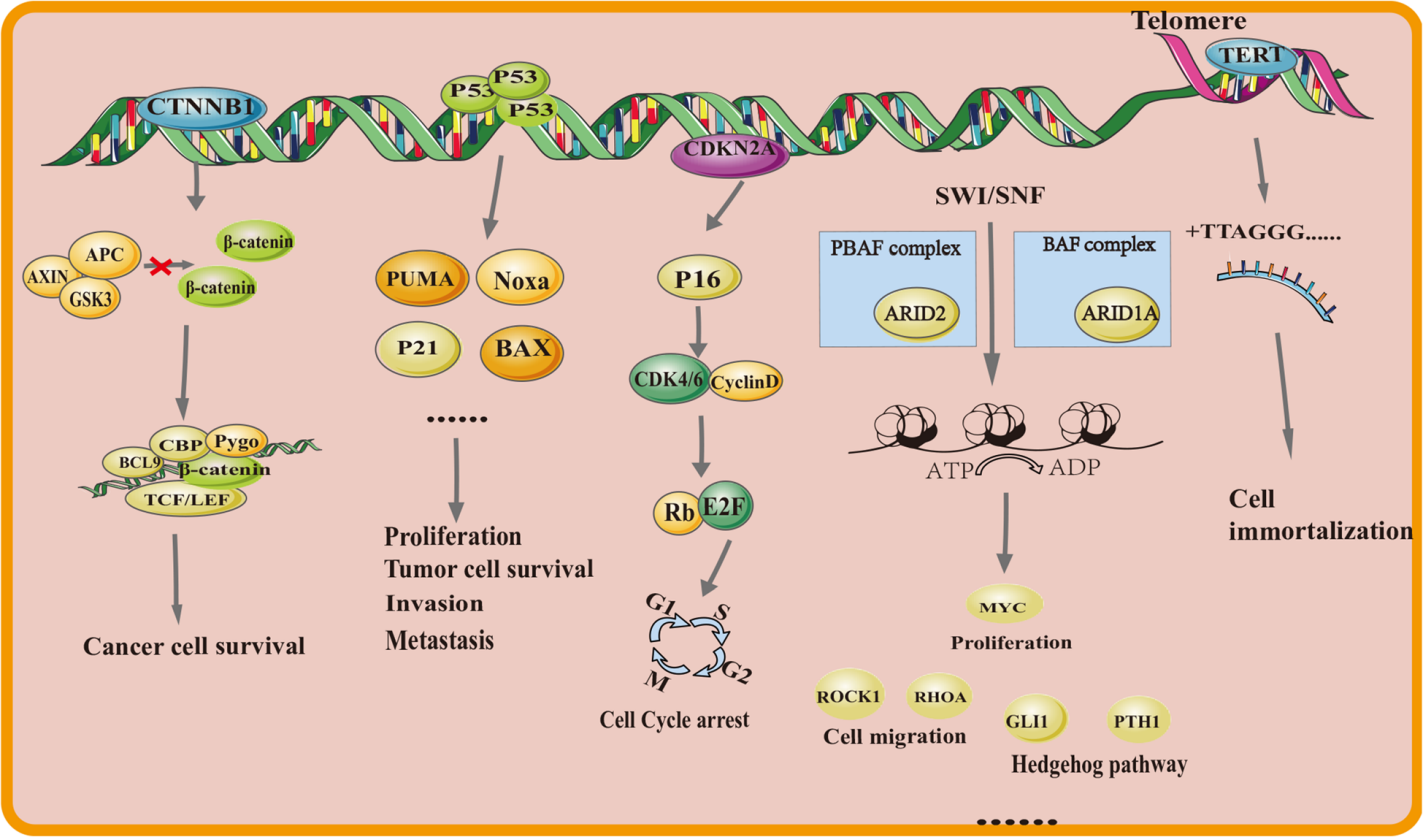
High frequency genetic markers of hepatocellular carcinoma and the key pathways

The tumorigenesis and development of HCC involve many complex genes and signaling pathways [57]. To date, the description of the genomic landscape of HCC patients at an early stage is mainly derived from the excised surgical tissues, and the recurrent genomic alterations are TERT, TP53, CDKN2A, CTNNB1, AXIN1, ARID1A, ARID2, MLL2, NFE2L2, and KEAP1 [58]. The analysis of mutation detection in ctDNA found that 27 of 48 pre-operative samples of patients in an early stage had at least one mutation in TP53, CTNNB1, and TERT [59]. It has been reported that TERT promoter (51%), TP53 (32%), CTNNB1 (17%), PTEN (8%), AXIN1 (6%), ARID2 (6%), KMT2D (6%), and TSC2 (6%) were prevalent in ctDNA analysis of 121 advanced HCC patients according to targeted ultra-deep sequencing [60]. Notably, mutations in TP53 and CTNNB1 were excluded. Using ddPCR targeting detection, at least one of the recurrent mutated loci situated in TP53 c.747G > T (p.R249S), CTNNB1 c.121A > G (p.T41A), CTNNB1 c.133 T > C (p.S45A), and TERT c.-124C > T have been detected in the peripheral blood of HCC patients, without being detected in normal HCC tissues or mononuclear cells of blood samples [61]. Studying the biological mechanisms of the aforementioned mutated genes in tumorgenesis and progression (Fig. 3) is conducive to screening of targeted populations and selection of therapeutic drugs, thus providing broad prospects for the clinical application of precision medicine (Table 3).

**Table 3:** Common biomarkers of ctDNA for HCC

Targeted genes	Classification	Description of alteration	Positive Rate	Relevant pathway	Main finding	Potential blockade agent	Reference
TP53	Suppressor	Inactivating mutation/ Homozygous deletion	32%	P53 signaling pathway	Patients more likely to have high AFP values or high HBV virus loads and increased quantities of hepato-carcinogenic risk factors, in addition to a poor prognosis	Bevacizumab, Wee1 inhibitors	[60, 62–64]
TERT	Oncogene	Promoter mutation/ amplification/ translocation	51%	Telomere maintenance	Patients are more prone to suffer from vascular invasion, an advanced TNM stage (*p* < 0.0001), large intrahepatic tumor size, high des-gamma carboxyprothrombin value, and increased mortality	GRN163L, BIBR1532, or some RNA interference	[60, 65, 66]
CTNNB1	Oncogene	Activating mutation	17%	WNT signaling pathway	Mutated CTNNB1 will help to stimulate gene expression, causing cell proliferation, anti-apoptosis and angiogenesis	Small-molecular blockades LGK874, OMP-54F28 …	[60, 67, 68]
AXIN	Suppressor	Inactivating mutation/ Homozygous deletion	6%	WNT signaling pathway	As a member of the core component of the “β-catenin destruction complex”	Small molecular blockade XAV939	[60, 69]
CDKN2A	Oncogene	Inactivating mutation/ Homozygous deletion	7%	Cell cycle	The mutation is correlated with an advanced stage and aggressive biological behaviors	Palbociclib	[70]
ARID1A	Suppressor	Inactivating mutation	14.3%	SWI/SNF complex related pathway	There is a dual role of the ARID1A gene in tumorigenicity and cancer suppression for different temporal and cellular background in HCC	–	[71]
ARID2	Suppressor	Inactivating mutation	6%	SWI/SNF complex related pathway	There is a dual role of the ARID2 gene in tumorigenicity and cancer suppression for different temporal and cellular background in HCC	–	[60]
RASSF1A	Oncogene	Methylation	36%^(47)^	MAPK/RAS signaling pathway	RASSF1A were shown to be closely related to HCC initiation and progression	–	[72]
SEPT9	Suppressor	Methylation	94.1%^(48)^	Cell division	SEPT9 are associated with early detection and poor prognosis of HCC tumors	–	[73]

#### TP53

The TP53 gene is closely related to the p53 signaling pathway and aberrations are involved in many biological regulation processes, generating increased proliferation, active epithelial-to-mesenchymal transition, and increased angiogenesis. There are > 120 various alterations and monitoring the *TP53* codon 249 mutation is particularly significant for clinical practice [74]. A recent study showed that the diagnostic ability of TP53 c.747G > T (p.R249S) detected in ctDNA led to positive outcomes in > 20% HCC patients, in contrast to only 3–4% of patients with pancreatic and gastric cancer, but was not detected in any of the healthy controls [75]. The presence of R249S in ctDNA reveals patients more likely to have high AFP values or high HBV virus loads and increased quantities of hepato-carcinogenic risk factors, in addition to a poor prognosis [76, 77]. Anti-angiogenesis drugs, such as bevacizumab or Wee1 inhibitors, can be used as inhibitors [62–64].

#### TERT

A mutation in the promoter region of the TERT gene always occurs early during HCC oncogenesis and is regarded as a driver gene for HCC carcinogenesis [78]. The expression of mutational TERT genes results in telomeres extending compensates for eroded telomeric ends and allows for epithelial cell immortalization [79]. Frequent occurrences of TERT promoter mutations located at − 124 and − 146 bp relative to the start codon in various cancers, especially alterations in -124C > T, clearly boost transcriptional activity in HCC cell lines [80]. Furthermore, patients with this type of mutation in ctDNA are more prone to have vascular invasion (*p* = 0.005) and are positively correlated with an advanced TNM stage (*p* < 0.0001), large intrahepatic tumor size (*p* = 0.05), high des-gamma carboxyprothrombin value (*p* = 0.005), and increased mortality [81, 82]. Telomerase-targeting compounds, like GRN163L, BIBR1532, or compounds that interfere with RNA, can decrease telomere length, which is expected to be applied in the following treatment, but still needs clinical evaluation [65, 66].

#### CTNNB1 and AXIN

CTNNB1 and AXIN are the key genes involved in the WNT/β-catenin pathway [83, 84]. Mutated CTNNB1 produces mutated β-catenin, which can escape phosphorylation and degradation. Negative regulation of mutated AXIN1or APC prevents the destruction complex from functioning, thus accelerating accumulation of β-catenin [85, 86]. The overaccumulation β-catenin will promote tumorigenesis or cancer progression. Analysis of CTNNB1 mutations (c.121A > G, c.133 T > C) had a frequency of 17% in ctDNA, while the positive rate was 6% for AXIN1 [60]. The expression of those genes can function as a compound tumor promoter involved in the progression of HCC based on an analysis of HCC tissue samples, which is consistent with the findings of a targeted sequence analysis of ctDNA [87, 88]. The recently reported small molecule blockade that aimed at attacking WNT ligands or receptors, such as LGK874 and OMP-54F28, preventing β-catenin degradation, such as NSAIDs, or inhibiting β-catenin from interacting with nuclear transcription, such as vitamin D and CWP232291, to block the WNT signaling pathway, are still in phase I or II clinical trials [67–69].

#### CDKN2A

Inactivation of the cyclin-dependent kinase inhibitor, CDKN2A, emerges in 7% of advanced HCC patients based on digital ctDNA sequencing and leads to overexpression of CDK4/6 [89]. With mutated CDKN2A, the upregulated CDK4/6 accelerates the G1/S phase transition in the cell cycle through thee CDK4/6-Rb-myc pathway and eventually promotes cell proliferation [70]. Additionally, patients with CDKN2A silencing correlate with an advanced stage and aggressive biological behavior [90]. Thus, CDK4/6 inhibitors leading to cell cycle arrest and cell death induction, such as palbociclib, ribociclib, and abemaciclib, can provide an effective target treatment for HCC patients with CDKN2A loss of function [70].

#### ARID1A and ARID2

*ARID1A* and *ARID2* are crucial components of the switch/sucrose non-fermentable (SWI/SNF) complex, an adenosine triphosphate-dependent complex participating in gene transcription stimulation or suppression via chromosomal remodeling [91]. Inactivated mutations of *ARID1A* or *ARID2* frequent present in many HCC patients and are clinically associated with cancer development [92]. Although ARID1A is found in 14.3% of the target HCC population and 6% for *ARID2* through ctDNA analysis [60, 71]. Inhibitors targeting the mutated SWI/SNF complex warrant further investigation.

### Altered methylations in ctDNA

Greater than 98% of methylation reactions occur on the cytosine of 5′-cytosine-phosphate-guanine-3′ (CpG) dinucleotide and catalyzed by DNA methyltransferase [93]. Previous studies showed that focal hypermethylation changes can drive inactivation of key tumor suppressor genes, dysregulation of regulatory regions that control cell cycle and growth, or reduced response to therapy. Hypomethylation of some gene sequences also occurs during the HCC-promoting process [94]. Abnormal epigenetic aberration of DNA methylation often occurs before tumor formation or development and can be considered as early tumor biomarkers for diagnosis or the identification tool to discriminate people at high risk of developing cancer [95]. Additionally, cancer type-specific methylation signatures displayed in different samples can help to identify the cancer tissue of origin, for tumor cells originating from different tissue types may share similar genotypes but exhibit a unique methylation profile [96, 97].

#### Hypermethylated changes

Villanueva et al. [98] reported that the DNA methylation aberrant landscape of HCC is depicted by the prevalence of RASSF1A, APC, NEFH, IGF2, SEPT9, and EFNB2. In an analysis of cfDNA, hypermethylation of p15, p16, APC, SPINT2, SFRP1, TFPI2, GSTP1, and RASSF1A were shown to be closely related to HCC initiation and progression [72]. The value of SEPT9 promoter methylation detected in cfDNA was also emphasized by Oussalah et al. [73] for it can discriminate HCC from cirrhotic patients with an area under the receiver operating characteristic curve (AUC) of 0.944. Additionally, Lu et al. [99] demonstrated that hypermethylation of RASSF1A, COX2, and APC genes detected in ctDNA can identify those HCC patients with negative AFP levels and is associated with greater susceptibility to tumor recurrence and poor survival prognosis. Recently, methylation status in ctDNA detected by a panel of several methylation sites has been considered to be a promising tool. For example, a panel of 10 DNA methylation markers was constructed and validated with high diagnostic sensitivity and specificity, in addition to a close relationship to tumor burden and clinical outcomes (*p* < 0.001) [100]. It has also been shown that a panel of 6 methylated DNA markers tested in a phase I pilot study and validated in a phase II clinical cohort study had a sensitivity of 95% and a specificity of 92% when HCC was detected among high-risk controls (AUC of 0.94) [101].

#### Hypomethylated changes

DNA hypomethylation may be involved in HCC through many mechanisms, including destabilization of chromosomes, repression free of imprinted genes, aberrant epigenetic expression, and activation of retrotransposition [102]. It is reported that ctDNA assay of the hypomethylation level nearby HBV integration sites can serve as an early detection tool for HCC [103]. Other genes, such as CTCFL promoters and UBE2Q1, the hypomethylation status of which in ctDNA are also believed to be relevant to the diagnostic and monitoring period for HCC patients [104, 105].

To conclude, assessment of ctDNA derived from peripheral blood samples may facilitate the diagnosis, staging, and surveillance of HCC, and offers signaling pathway inhibitors or targets for precision therapy based on the specific mutation identified.

## Diagnostic value of ctDNA for HCC patients

### Early diagnosis of HCC

Before the genetic and epigenetic landscape of HCC was well defined, many studies investigated quantitative changes in cfDNA levels to achieve the goal for early detection of HCC [106, 107]. The cfDNA levels, however, can also increase with exercise, inflammation, surgery, or tissue injury in healthy individuals [14], leading to an application limitation.

Cancer-related sequences detected by liquid biopsy are often well-known mutations that have already shown clinical relevance, which limits the application of mutated genes in early tumor diagnosis [59, 108]. Cohen et al. (29) combined mutations in ctDNA and circulating proteins for several types of common cancers for early detection of tumors (Cancer SEEK). For HCC, the sensitivity was nearly 95% and it detected nearly 100% of HCC patients in an early stage (stage I) among a cohort of 44 patients with liver cancer [75].

In contrast to mutational sequences in ctDNA, the genome-wide distribution of numerous, densely clustered DNA methylation aberrations significantly impact robust cancer detection and high sensitivity in cancer diagnostics. Aberrant methylation in the promoter region is always involved in the initiation of HCC [109]. Based on previous findings, ctDNA positivity precedes imaging findings and prior to positive AFP values [99, 110]. Analysis of methylation alterations in ctDNA has been reported to accurately distinguish early-stage HCC patients (BCLC stage 0/A) from non-HCC and high-risk patients with a history of HBV infection or liver cirrhosis [111]. Chen et al. [112] also described an assay interrogating cancer-specific methylation signals in ctDNA, and exhibted the potency of early diagnosis among 5 types of tumor types (liver cancer contained), outperforming conventional diagnosis by up to 4 years. These results suggest the feasibility of ctDNA as an early-onset biomarker for HCC detection.

Wong et al. [113] first reported the positive rate of methylation in p15 and p16 is 48% and the rate of p15/p16 detection can be as high as 92%. RASSF1A was also confirmed to have a valuable role in the early diagnosis of HCC by Mohamed et al. [114], with a sensitivity of 90%. Moreover, in their study, RASSF1A can also discriminate HCC patients from healthy patients with a predictive accuracy of 77.5% based on logistic regression analysis, and it can also differentiate HCC and hepatitis C patients with an area under the receiver operating characteristic curve (AUC) value of 0.733 nmol/L and predictive accuracy of 72.5%. In another study conducted by Xu et al. [100], a 10-methylation marker panel was constructed as a diagnostic prediction model, with a sensitivity of 85.7% and a specificity of 94.3% for HCC patients in the training cohort, and a sensitivity of 83.3% and a specificity of 90.5% in the validation cohort. Thus, the combined detection of methylation status among multiple genes can effectively improve the diagnostic efficacy. Xu et al. [100] also pointed out that a combined prognostic score can differentiate high-risk liver disease and HCC. Similarly, A 32-gene diagnostic model was developed by Cai et al. [111], which had superior performance in distinguishing early HCC or small tumors (≤ 2 cm) from non-HCC compared to AFP (AUC = 88.4; 95% CI: 85.8–91.1%). The model can discriminate HCC from chronic HBV or cirrhosis.

### Etiologic diagnosis for HCC

In addition, several investigations have shown that multiple carcinogens for HCC, such as chronic HBV or HCV virus infection, alcohol abuse, NAFLD/NASH, and aflatoxin B1, may have different somatic mutations [115]. Based on an analysis of the correlation between tumor tissues and their carcinogens, mutations in the TERT promoter are prevalent in HCV-induced HCC, as well as the CTNNB1, ARID2, and GPC3 sequences [116]. Specific mutations in the HLA region, KIF1B, STAT4, GRIK1, ErbB2, TP53, and PTEN are mainly found in HCC caused by HBV [117], and the genomic aberration of HCC samples in the region of TP53 and GPCR subfamily members (ADGRB1, ADGRB2, and ADGRB3) are closely related to aflatoxin B1 [118]. In addition, changes in DNA sequences in patients with alcohol-related HCC have recurrent mutations in CTNNB1, TERT, ARID1A, SMARCA2, and PNPLA3 I148M [119]. Furthermore, patients with NAFLD/NASH are more prone to exhibit mutations in rare germline hTERT, and genes involved in calcium signaling, such as Sav, YAP, and TAZ [120].

It has been suggested that detection of specific genomic aberrations in ctDNA will distinguish HCC types and facilitate individualized treatment of HCC patients; however, retrospective studies have demonstrated that patients with TERT promoter mutations in ctDNA are closely related to HCV infection [121] and ERBB2 alterations within ctDNA are more likely to be identified with characteristics of HBV infection [74], thus confirming the possibility of identifying HCC subtypes by ctDNA.

## Monitoring response to therapy by ctDNA

### Response to targeted therapy

Thus far, no identical genomic profile has been detected, suggesting that analyzing the mutational genomic landscape of a patient can enable customized treatment. For example, Ikeda et al. [89] evaluated 14 patients using a commercial NGS panel and showed that advanced HCC patients with PTEN-inactivating and MET-activating mutations can benefit from therapy with sirolimus and cabozantinib, which are inhibitors of the relevant pathways. Patients with CDKN2A-inactivating and CTNNB1-activating mutations who received palbocilib (a CDK4/6 inhibitor) and celecoxib (a Cox-2/Wnt inhibitor) subsequently had decreased AFP levels.

In addition, the gene sequence of ctDNA may change as the pressure of treatment changes. Theoretically, ctDNA shares the same tumor genetic information with primary tumor cells from which they originated and represent a real-time biomarker due to the rapid clearance [14]. In a recent study, ctDNA of a patient treated with capecitabine was profiled at multiple time points and displayed a decrease in initial ARID1A and BRCA2 mutational alleles during systemic treatment and emergence of TP53 aberration after disease progression [89]. Making this speculation more powerful, Alunni-Fabbroni et al. [122] reported that the majority of genomic variants (68%) were discovered after the beginning of sorafenib treatment, the first-line targeted therapy for advanced HCC patients [123], indicating that treatment alone may affect the selection of gene cloning. Additionally, ctDNA can also serve as an eligible tool for evaluating the treatment efficiency of refametinib monotherapy and refametinib plus sorafenib combined therapy in advanced HCC patients with a mutational RAS allele [124]. Above all, it is speculated that we can monitor disease progression of HCC patients and make timely response treatment measures by analyzing the ctDNA genomic profile serially.

The molecular ctDNA may also serve as a biomarker for predicting drug resistance. DNA methylation alterations in cell lines can actuate EMT-mediated resistance to sorafenib in HCC patients at an advanced stage [125]. This non-invasive method of obtaining genomic drug resistance information avoids the difficulty of re-obtaining and analyzing biopsy tissues of advanced HCC patients.

There are some biologic and technique limitations that need to be addressed. Due to the complexity of the signal interaction network, the tumor microenvironment, and diverse genetic backgrounds, HCC has high tumoral heterogeneity [126]. Thus, a single biomarker may be inadequate for personalized medicine selection. Moreover, the low incidence of the potential predictive biomarkers, as shown in Table 3, makes it difficult to drive further clinical trials. The task appears to be more daunting for the presence of comorbid cirrhosis in most patients with HCC because drug-related toxicity would be another limitation. A consensus of standard operating procedures to ensure accuracy of ctDNA test has not been achieved. Current available methodologies are time-consuming and costly, and most have insufficient sensitivity and cannot cover the entire genomic loci [16].

### Response to immunotherapy

Immunotherapy, which can be represented by immune checkpoint blockade (ICB), has transformed clinical practice in cancer treatment. At present, ramucirumab is recommended as second-line medication after sorafenib for advanced HCC patients with serum AFP levels ≥400 ng/mL [127]. And a large phase III study (IMbrave 150) reported that atezolizumab plus bevacizumab improves prognostic outcomes superior to sorafenib [128]. On 29 May 2020, the combination of atezolizumab and bevacizumab for the treatment of unresectable or metastatic HCC patients was approved by the Food and Drug Administration [129]. Despite the initial successes achieved with ICB systemic therapy, patients suitable for immunotherapy need to be identified using molecular assays.

Previous studies have proposed that ctDNA can be used to measure the tumor mutation burden, referring to the total number of alterations per mega-base in a specific exon region of tumor genomic sequences and identify tumor patients who have a high likelihood of response to immunotherapy [130, 131]. This response can be detected 38 days earlier than the radiographic response [132]. Moreover, ctDNA can differentiate the true progress from pseudo-progression caused by inflammation from ICB therapy [133] and alterations in some specific genes may be related to immune-related adverse events [134]. Relevant studies have mainly focused on melanoma, non-small cell lung cancer, and gastric cancer [134–136]. There is still a gap in clinical research of ctDNA in ICB therapy for HCC patients because mutational DNA molecules in the HCC population have not been pre-defined and those aberrations which exist in HCC can also be detected in benign hepatic diseases [137]. These deficiencies can be overcome by establishing a panel consisting of HCC-associated genetic aberrations for sequencing assay.

### Response to surgical therapy

Hepatic resection, liver transplantation, and local ablation have been suggested to be the standard curative treatment method for early-stage HCC patients [138, 139]. Nevertheless, the postoperative recurrence rate remains at a high level of > 60% HCC patients within 5 years [140]. A considerable number of post-operative patients may have occult micrometastases or minimal residual disease (MRD) without clinical or radiologic signs; however, ctDNA can serve as a biomarker to detect MRD.

In a recent study, 34 HCC patients underwent surgical resection followed by other adjuvant therapies during the follow-up period in China underwent ctDNA detection using NGS-based technology [110]. The study indicated that ctDNA identified 10 of 17 patients had a recurrence within 1 year, prior to serum protein biomarker detection. One patient with consistent ctDNA-positivity had a recurrence on day 610, suggesting that he had MRD for a relatively long period. Further discussion of their study suggested that patients with ctDNA-positivity were thought to be at high risk for recurrence and metastasis (log-rank, *p* < 0.001) by Kaplan–Meier analysis. Another study relied on ddPCR technology to identify four hot-spot mutants (TP53-rs28934572, TRET-rs1242535815, CTNNB1-rs121913412, and CTNNB1-rs121913401) and arrived at a similar conclusion that specific aberrations displayed in ctDNA can be defined as an independent risk factor of HCC patients for post-operative recurrence [88]. Moreover, ctDNA can track longitudinal changes of different mutants and therapeutic responses in real-time monitoring. For example, Cai et al. [141] reported a patient monitored with a somatic mutation (HCKp.V174M). The alteration was detected after the first TACE treatment, and then became undetectable after the second hepatic surgery and sharply increased during the second recurrence. In summary, the somatic mutation frequency of ctDNA is capable of detecting a recurrence in advance, unlike traditional imaging tests and protein biomarkers.

## Predictive value of ctDNA for prognosis

ctDNA levels have been reported to be closely correlated with tumor burden, cell proliferation, and Edmondson grade [142]. It has been reported that patients with a high level of ctDNA are more likely to have metastases and worse survival outcomes [122]. Nevertheless, the practicability of this research with only 13 patients as entities in the study cohort requires further verification.

In contrast, targeted ctDNA analysis of intra-tumoral heterogeneity enables prediction of survival outcomes among HCC patients. TERT promoter mutations were the recurrent point mutations and aberrant alteration of TERT C228T has been shown to be associated with increased mortality when detected in ctDNA [81, 121]. Alterations in other driver genes, such as TP53 and CTNNB1, also have a negative performance for prognosis [108, 143]. MLH is a pivotal gene for mismatch repair during DNA replication and the defections impact genomic instability and cancer development [144]. The presence of the alteration in MLH1 chr3:37025749 T > A exhibited a worse survival rate [145].

Recently, Li et al. [146] also reported that promotor methylation of insulin-like growth factor binding protein 7 (IGFBP7) in the cfDNA is an independent risk factor for 155 patients undergoing surgical resection, indicating that continuous detection of tumor-specific driver gene mutations and methylation in ctDNA can be unrestricted by genetic heterogeneity and achieve the prediction goal of HCC.

## Limitations and future perspective

Although the potential application of ctDNA is promising for monitoring the occurrence, development, and prognosis of HCC, there is still controversy regarding the clinical utility.

First, the expression of non-DNA based alterations, including hormone receptors or other proteins, cannot be identified by ctDNA analysis, which also plays a significant role in the diagnosis and treatment strategies of tumors. Thus, the histologic information carried by ctDNA is incomplete. Second, analysis of clinical relevance of ctDNA concentrated on systemic therapy and hepatectomy, lacking investigations in transarterial chemoembolization (TACE), selective internal radiation therapy (SIRT), locational ablations and immunotherapy, leading to insufficient evidence supported the clinical utility. Nevertheless, a considerable number of clinical trials registered with clinicaltrials.gov and some selected patents, as presented in Tables S1 and S2, respectively, highlight the potency of ctDNA in HCC management. Third, a universal tool with high sensitivity and specificity to ensure the accuracy of research results is urgently needed for clinicians. Although the detection of ctDNA mutations and methylation have been successfully applied in advanced common cancers, 15% of patients with metastatic cancer may not have sufficient ctDNA levels to allow for mutational profiling from plasma [147]. It should also be mentioned that most data are from Asian countries and with restricted samples. Thus, these data have limited generalizability.

To select the most appropriate mutation profiling specimens guiding clinical-decision making, it is necessary to perform a comparative study of CTC-derived DNA (CTC-DNA), ctDNA, and tumor tissue DNA (tumor DNA; Table 4). Sundaresan et al. [148, 149] believe the overall performance of ctDNA is superior to CTCs, but there is still a 20–30% blank that needs to be covered by combination analysis of ctDNA and CTC-DNA in non-small-cell lung cancer, a finding also corroborated in colorectal cancer [150], thoracic cancer [151], metastatic prostate cancer [152], and HCC [153].

**Table 4:** Comparison of CTC-DNA, ctDNA and tumor DNA

	Advantages	Disadvantages
**CTC-DNA**	Greater allele frequency New mutations	Lower coverage depth Less abundant
**ctDNA**	Abundant analysis materials High sensitivity	Lower allele frequency Dying cells source
**Tumor DNA**	Gold standard	Non-enough analysis material Unable to reflect tumor heterogeneity Risk and discomfort

On the other hand, some researchers argue that matching genomic biomarkers with systematic therapies is not sufficient. First, greater than half of the patients do not have actionable alterations. Second, there is a wide range of non-genetic factors relevant to oncogenesis and progression, and some mutations may result in different responses to the same drugs in different cancers. Moreover, the identification of splice variants rely on mRNA analysis instead of genomic NGS. The immunoassays are also closely related to RNA-based analysis [154]. Furthermore, with advances in technologies, it is possible to conduct RNA analysis of a single CTC [155]. Jan et al. [156] demonstrated that tissue -based RNA profiling can be transferred to CTC-RNA expression analysis and the expression can provide guidance on treatment selection. Some researchers have also reported an increased match rate by incorporation of transcriptomic data [157, 158]. Thus, it is hypothesized that combining ctDNA and CTC-RNA data may improve the predictability of the treatment response. Although there is no definitive answer to this important question, it emphasizes the desired direction of research in liquid biopsy (multi-parametric co-analysis) to facilitate the development of precision oncology.

Last, the currently used methodologies require full preparation of biological material and expensive specialized laboratory equipment, increasing the difficulty in popularization.

## Conclusion

In conclusion, ctDNA is a transformative biomarker aimed at precision monitoring of HCC patients during the overall course of treatment. Superior performance in initial diagnosis, optimal selection of relevant targeted therapy or immunotherapy, and a timely decision of the need to transform therapeutic strategies are of great significance for improving the survival outcome of HCC patients and development of precision oncology for HCC. Because the choice of ctDNA markers has not yet reached agreement and detection technology is time consuming and expensive, ctDNA analysis should be further explored when applied to patients with HCC.

## Supplementary Information

**Additional file 1: Supplementary Table S1.** Current trials registered with clinicaltrials.gov exploring ctDNA in hepatocellular carcinoma.

**Additional file 2: Supplementary Table S2.** Recent patents of ctDNA analysis in hepatocellular carcinoma.

## Data Availability

Not applicable.

## Abbreviations

CTNNB1: Cadherin-associated protein beta 1
AXIN: Axis inhibition
AFP: Alpha-fetoprotein
TERT: Telomerase reverse transcriptase
TP53: Tumor protein p53
CDKN2A: Cyclin-dependent kinase inhibitor 2A
ARID1A: AT-rich interaction domain 1A
MLL2: Mixed-lineage leukemia 2
NFE2L2: Nuclear factor, erythroid 2 like 2
KEAP1: Kelch-like ECH-associated protein 1
KMT2D: Lysine methyltransferase 2D
TSC2: TSC complex subunit 2
RASSF1A: Ras association domain family member 1
NEFH: Neurofilament heavy
IGF2: Insulin-like growth factor 2
SEPT9: SEPTIN9
TFPI2: Tissue factor pathway inhibitor 2
GSTP1: Glutathione S-transferase pi 1
SMARCA2 – SWI/SNF related: matrix-associated, actin-dependent regulator of chromatin, subfamily a, member 2
PNPLA3: Patatin-like phospholipase domain containing 3
ERBB2: Erb-b2 receptor tyrosine kinase 2
PTEN: Phosphatase and tensin homolog
BRCA2: Breast cancer 2
RAS: Resistance to audiogenic seizures
IGFBP7: Insulin-like growth factor binding protein 7
NAFLD: Non-alcoholic fatty liver disease
NASH: Non-alcoholic steatohepatitis
VEGFR: Vascular endothelial growth factor receptor
PDGFR: Platelet-derived growth factor
RASSF1A: Ras association (RalGDS/AF-6) domain family member 1
COX2: cytochrome c oxidase subunit II
CTCFL: CCCTC-binding factor like
UBE2Q1: ubiquitin conjugating enzyme E2 Q1
BCLC stage: Barcelona Clinic Liver Cancer stage
