# Correction to: Current status of ctDNA in precision oncology for hepatocellular carcinoma

**DOI:** 10.1186/s13046-021-02032-3

**Published:** 2021-07-13

**Authors:** Yan Li, Yuanyuan Zheng, Liwei Wu, Jingjing Li, Jie Ji, Qiang Yu, Weiqi Dai, Jiao Feng, Jianye Wu, Chuanyong Guo

**Affiliations:** 1 Department of Gastroenterology, Putuo People’s Hospital, Tongji University School of Medicine, number 1291, Jiangning road, Putuo, Shanghai, 200060 China; 2 Department of Gastroenterology, Shanghai Tenth People’s Hospital, Tongji University School of Medicine, Number 301, Middle Yanchang road, Jing’an, Shanghai, 200072 China

**Correction to: J Exp Clin Cancer Res 40, 140 (2021)**

**https://doi.org/10.1186/s13046-021-01940-8**

Following publication of the original article [1], the authors identified an error in the author affiliations. Chuanyong Guo’s first affiliation was missing; Chuanyong Guo’s affiliations are therefore:

- ^1^Department of Gastroenterology, Putuo People’s Hospital, Tongji University School of Medicine, number 1291, Jiangning road, Putuo, Shanghai, 200060, China
- ^2^Department of Gastroenterology, Shanghai Tenth People’s Hospital, Tongji University School of Medicine, Number 301, Middle Yanchang road, Jing’an, Shanghai 200072, China

The original article has been corrected.
