# Supplementary material for: Current status of ctDNA in precision oncology for hepatocellular carcinoma
Source: J Exp Clin Cancer Res. 2021 Apr 26;40:140. doi: 10.1186/s13046-021-01940-8 (PMC8074474; doi:10.1186/s13046-021-01940-8)
Supplement: Supplementary file 1 — Additional file 1: Supplementary Table S1. Current trials registered with clinicaltrials.gov exploring ctDNA in hepatocellular carcinoma. [file 13046_2021_1940_MOESM1_ESM.docx]

**Supplementary Table S1** Current trials registered with clinicaltrials.gov exploring ctDNA in hepatocellular carcinoma

| Trial number | Trail name | Focus/goals | Study type | Estimated completion | status |
| --- | --- | --- | --- | --- | --- |
| NCT03483922 | HCC Screening Using DNA Methylation Changes in ctDNA | To develop and test non-invasive biomarkers based on methylation changes in PBMC, T-cells and circulated tumor DNA in hepatocellular carcinomas patients. | Prospective observational cohort | September 1, 2020 | Recruiting |
| NCT03839706 | Relationship Between 18FDG PET/MRI Patterns and ctDNA to Predict HCC Recurrence After Liver Transplantation | The Investigators will assess the accuracy of 18F-FDG PET/MRI and ctDNA as a tool to predict HCC recurrence after liver transplant. | Interventional (Clinical Trial) | December 2022 | Recruiting |
| NCT04111029 | Liquid Biopsy in Hepatocellular Carcinoma (HCC Gene Panel | Use of 'liquid biopsy' by assessing circulating cell free DNA enables the clinician to offer targeted immunotherapy or signaling pathway inhibitors. | Prospective observational cohort | October 2021 | Not-yet recruiting |
| NCT04506398 | Heterogeneity and Evolution of hepatocellular Carcinoma in Post-transplant HCC Recurrence (HELP-2020) | This study will evaluate the heterogeneity and evolution pathway between primary HCC and tumor relapse after liver transplant. | Prospective observational cohort | August 2022 | Not yet recruiting |
| NCT02838836 | Tumor Cell and DNA Detection in the Blood, Urine and Bone Marrow of Patients With Solid Cancers | CTCs/DTCs and cfDNA isolated from cancer patients will be characterized for genetic alterations and expression of key signaling/proliferation biomarkers and grow in vivo in nude mice. | Prospective observational cohort | June 1, 2022 | Recruiting |
| NCT04484636 | PLATON - Platform for Analyzing Targetable Tumor Mutations (Pilot-study) (PLATON) | In a first approach PLATON's pilot-study assesses genomic profiling in gastrointestinal cancer therapy and the frequencies of targetable mutations including Tumor Mutational Burden (TMB) and Microsatellite Instability Status (MSI), performing Next-generation deep sequencing (NGS) using the Foundation Medicine assays on tumor specimen and EDTA-whole blood samples. | Prospective observational cohort | July 30, 2021 | Not yet recruiting |
| NCT03245190 | Study of Chiauranib in Patients with Advanced Hepatocellular Carcinoma | This clinical trial is studying the efficacy and safety of chiauranib works in treating patients with advanced hepatocellular carcinoma, in the meantime, exploring the latent biomarkers accompany with chiauranib, as well as the relevancy of which and clinical benefit. | Interventional (Clinical Trial) | December 2019 | Recruiting |
| NCT04134559 | Checkpoint Inhibition in Pediatric Hepatocellular Carcinoma | In this research study, the investigators plan to investigate whether pediatric patients with hepatocellular carcinoma experience stable disease or response to pembrolizumab and to explore different biological factors of the tumor and immune system that might help us predict whether pediatric patients with HCC may benefit from treatment with pembrolizumab. | Interventional  (Clinical Trial) | January 1, 2023 | Recruiting |
| NCT04358185 | Itacitinib in Advanced Hepatocellular Carcinoma (JAKaL) | Itacitinib in advanced hepatocellular carcinoma (JAKaL) and assessment of presence of predefined JAK1 mutations in ctDNA | Interventional   (Clinical Trial) | December 31, 2021 | Recruiting |
