# Supplementary material for: Current status of ctDNA in precision oncology for hepatocellular carcinoma
Source: J Exp Clin Cancer Res. 2021 Apr 26;40:140. doi: 10.1186/s13046-021-01940-8 (PMC8074474; doi:10.1186/s13046-021-01940-8)
Supplement: Supplementary file 2 — Additional file 2: Supplementary Table S2. Recent patents of ctDNA analysis in hepatocellular carcinoma. [file 13046_2021_1940_MOESM2_ESM.docx]

**Supplementary Table S2** Recent patents of ctDNA analysis in hepatocellular carcinoma

| Patent number | Year | Inventors | Patent title | Main finding | Reference |
| --- | --- | --- | --- | --- | --- |
| [CN108949980 (A)](https://worldwide.espacenet.com/publicationDetails/biblio?DB=EPODOC&adjacent=true&locale=cn_EP&FT=D&date=20181207&CC=CN&NR=108949980A&KC=A) | 2018 | Liao et al. | Kit for detecting HCC | The present invention consisting of 5 pairs of primers for detecting 5 methylation specific sites in the genome, which used for early diagnosis and mass screening of HCC. | [1] |
| [WO2020007089 (A1)](https://worldwide.espacenet.com/publicationDetails/biblio?DB=EPODOC&adjacent=true&locale=cn_EP&FT=D&date=20200109&CC=WO&NR=2020007089A1&KC=A1) | 2020 | Jiao et al. | Construction and sequencing data analysis method for ctDNA library for simultaneously detecting various common mutations in liver cancer | A construction and sequencing data analysis method for a ctDNA library for simultaneously detecting various common mutations in HCC. | [2] |
| [CN109943637 (A)](https://worldwide.espacenet.com/publicationDetails/biblio?DB=EPODOC&adjacent=true&locale=cn_EP&FT=D&date=20190628&CC=CN&NR=109943637A&KC=A) | 2019 | Liu et al. | Liver cancer diagnosis and prognosis evaluation system based on circulating tumor DNA mutation detection | The present invention includes a detection module, a filtering module, an analysis module, a summarization module and a display module, connected in sequence, constructing a ctDNA based HCC diagnosis and prognosis evaluation system | [3] |
| [CN105950750 (A)](https://worldwide.espacenet.com/publicationDetails/biblio?DB=EPODOC&adjacent=true&locale=cn_EP&FT=D&date=20160921&CC=CN&NR=105950750A&KC=A) | 2016 | Liu et al. | Genetic group and kit for liver cancer diagnosis and prognosis evaluation | The present invention relates to a gene cluster for diagnosis and prognosis assessment of HCC based on next-generation sequencing technology, and a gene test kit, designed for this gene cluster, for diagnosis and prognosis assessment of HCC. | [4] |
| [KR102152893 (B1)](https://worldwide.espacenet.com/publicationDetails/biblio?DB=EPODOC&adjacent=true&locale=cn_EP&FT=D&date=20200907&CC=KR&NR=102152893B1&KC=B1) | 2020 | Kim et al. | MLH1 DNA use for detection of hepatocellular carcinoma specific MLH1 circulating tumor DNA mutation | The invention relates to the detection of circulating tumor DNA variation of species-specific MLH1 gene of hepatocellular carcinoma. The invention is simple, effective, providing a composition capable of confirming HCC mutations with predictable prognosis. | [5] |
| [CN110387404 (A); CN110387404 (B)](https://worldwide.espacenet.com/publicationDetails/biblio?DB=EPODOC&adjacent=true&locale=cn_EP&FT=D&date=20191029&CC=CN&NR=110387404A&KC=A) | 2019 | Tang et al. | Magnetic bead-PNA probe compound and application thereof in enriching circulating tumor DNA in hepatocellular carcinoma | The invention discloses a magnetic bead-PNA probe compound and an application thereof in enriching ctDNA in HCC. The detection sensitivity on the ctDNA in HCC is improved by more than 10 times. | [6] |

**Reference**

1. Liao X DZ, Xiang Y, Zhang H, Huang F, Zhang Z, Li H, Yao A, Fan L, Li J, et al: **Kit for detecting hepatocellular carcinoma (HCC).** CN108949980A; 2018.

2. Jiao Y QC, Wang P, Chen K, Wang Y, Song Q，Wang S; Yan H: **Construction and sequencing data analysis method for ctDNA library for simultaneously detecting various common mutations in liver cancer.** WO2020007089A1; 2020.

3. Liu J，Liu X CZ, Chen G, Li Z, Dong X: **Liver cancer diagnosis and prognosis evaluation system based on circulating tumor DNA mutation detection.** CN109943637A; 2019.

4. Liu X LJ, Cai Z, Chen G, Dong X: **Genetic group and kit for liver cancer diagnosis and prognosis evaluation.** CN105950750A; 2016.

5. Kim SS CJ, Eun JW, Cho HJ: **MLH1 DNA use for detection of hepatocellular carcinoma specific MLH1 circulating tumor DNA mutation.** KR102152893B1; 2020.

6. Tang J ZZ, Tang J: **Magnetic bead-PNA probe compound and application thereof in enriching circulating tumor DNA in hepatocellular carcinoma.** CN110387404A; 2019.
